# Megaprosthesis in Non-Oncologic Settings—A Systematic Review of the Literature

**DOI:** 10.3390/jcm12124151

**Published:** 2023-06-20

**Authors:** Andrea Sambri, Stefania Claudia Parisi, Renato Zunarelli, Lorenzo Di Prinzio, Lorenzo Morante, Gianluca Lonardo, Marta Bortoli, Andrea Montanari, Roberto De Cristofaro, Michele Fiore, Massimiliano De Paolis

**Affiliations:** Orthopedic and Traumatology Unit, IRCCS Azienda Ospedaliero-Universitaria di Bologna, 40138 Bologna, Italy; stefaniaclaudiaparisi@hotmail.it (S.C.P.); renato.zunarelli@ior.it (R.Z.); lorenzo.diprinzio@ior.it (L.D.P.); lorenzo.morante@ior.it (L.M.); gianluca.lonardo@gmail.com (G.L.); marta.bortoli@ior.it (M.B.); andrea.montanari36@studio.unibo.it (A.M.); roberto.decristofaro@aosp.bo.it (R.D.C.); michele.fiore@ior.it (M.F.); massimiliano.depaolis@aosp.bo.it (M.D.P.)

**Keywords:** severe bone loss, megaprosthesis, non-oncologic, pseudoarthrosis, fracture

## Abstract

Modular megaprostheses (MPs) are commonly used after bone-tumor resection, but they can offer a limb salvage solution in massive bone defects. The aim of this systematic review of the Literature is to provide a comprehensive data collection concerning the use of MPs in non-oncologic cases, and to provide an overview of this topic, especially from an epidemiologic point of view. Three different databases (PubMed, Scopus, and Web of Science) were searched for relevant articles, and further references were obtained by cross-referencing. Sixty-nine studies met the inclusion criteria, reporting on cases of MP in non-oncologic cases. A total of 2598 MPs were retrieved. Among these, 1353 (52.1%) were distal femur MPs, 941 (36.2%) were proximal femur MPs, 29 (1.4%) were proximal tibia MPs and 259 (10.0%) were total femur MPs. Megaprostheses were most commonly used to treat periprosthetic fractures (1158 cases, 44.6%), in particular in the distal femur (859, 74.2%). Overall, complications were observed in 513 cases (19.7%). Type I (soft tissue failures) and type IV (infection) according to the Henderson classification were the most frequent (158 and 213, respectively). In conclusion, patients with severe post-traumatic deformities and/or significant bone loss who have had previous septic complications should be considered as oncologic patients, not because of the disease, but because of the limited therapeutic options available. The benefits of this treatment include relatively short operative times and immediate weight-bearing, thus making MP particularly attractive in the lower limb.

## 1. Introduction

Reconstruction of massive defects of long bones is a demanding surgical procedure that poses multiple challenges for the treating orthopedic surgeon [1]. Several clinical scenarios can be associated with significant bone loss, which is comparable to the resection of a bone tumor. These can include severe trauma, failed osteosynthesis with a non-union or periprosthetic fracture, and multiple revisions of arthroplasty for either an aseptic loosening or a periprosthetic joint infection (PJI) [2,3,4,5,6]. Patients frequently have undergone a number of previous procedures which may limit the options of reconstruction or may involve a number of comorbidities.

There are various reconstructive strategies to treat bone defects such as autograft and allogeneic bone grafting, bone transport, and the use of standard prosthesis and megaprosthesis (MP). Modular MPs are commonly used after bone-tumor resection, but they can offer a limb-salvage solution in such difficult-to-manage situations [7]. A major advantage of MPs is their intraoperative flexibility, which enables the surgeon to reconstruct huge bone defects [7,8,9].

However, MPs have inherent disadvantages including implant costs and a lack of further revision options, increased risk of dislocation, and PJI [2,9]. Megaprosthesis may be preferable in elderly patients with loose implants and insufficient bone stock or in patients who require short hospitalization and rapid recovery because of low activity levels and multiple comorbidities [10,11,12].

Moreover, in such cases, bone and soft tissue conditions are completely different from the oncological patient group. The knee extensor mechanism is very often in a critical condition, particularly in post-traumatic septic patients who have undergone multiple surgeries. Tissue adhesion, scar interference, muscular and tendon impairment, soft tissue retractions, osteoporosis, and skin problems can lead to a reduced function of the knee and severe joint stiffness, and also create adverse conditions during the reconstructive step [13,14].

The aim of this systematic literature review is to provide a comprehensive data collection concerning the use of MP in non-oncologic cases and to provide an overview of this topic, especially from an epidemiologic point of view.

## 2. Materials and Methods

This systematic review was conducted in accordance with the 2020 PRISMA guidelines (Preferred Reporting Items of Systematic Reviews) [15].

All studies (randomized controlled trials (RCTs), prospective (PCCS) and retrospective comparative studies (RCCS), prospective (PCS) and retrospective case series (RCS)) reporting the use of megaprostheses in non-oncologic cases were included. Biomechanical studies, cadaveric studies, “in vitro” studies, and animal model studies were excluded. Only articles in English published in a peer-reviewed journal were included. Articles published before 1995 and those reporting on MP for oncologic reconstructions were also excluded.

The criteria used to select articles allowed us to extrapolate data about the use of an MP in non-oncologic cases. Studies eligible for this systematic review were identified through an electronic systematic search of PubMed, Scopus, and Web of Science, up to 30 April 2023. The search string used was as follows: (megaprosthesis OR endoprosthetic replacement) AND (pseudoarthrosis OR non-union OR non-oncologic OR fracture OR infection OR periprosthetic infection OR loosening). Articles without an abstract were excluded from the study. The articles were screened considering the relevance of titles and abstracts and looking for the full-text article when the abstract provided insufficient information about inclusion and exclusion criteria.

Articles that were considered relevant via electronic search were retrieved in full text, and a cross-referencing search of their bibliographies was performed to find further related articles. Reviews and meta-analyses were also analyzed in order to broaden the search to studies that might have been missed through the electronic search. All duplicates were removed, and all the articles retrieved were analyzed. After the first screening, records without eligibility criteria were excluded.

Remnant studies were categorized by type, according to the Oxford Centre for Evidence-Based Medicine (OCEBM). 

Each study was assessed by two reviewers (SC.P. and R.Z.) independently and in duplicate; disagreement was resolved by the senior author (A.S.). All the included studies were analyzed, and data related to topics of interest were extracted and summarized. 

In detail, the data extracted included study type, mean age, site, indication to implant an MP, mean follow-up, complications, and functional outcomes. Complications that required subsequent revision of the prosthesis were recorded and classified according to Henderson et al. [16]. Functional outcomes were reported according to the reported scoring systems used in each study analyzed in this review. Only homogeneous series which included only one MP site were considered to assess cumulative data on indication to implant an MP, complications, and functional results.

The study is descriptive, and data are presented as total frequencies and percentages. The heterogeneity of most of the included studies did not allow any statistical analysis.

## 3. Results

**Figure 1:**
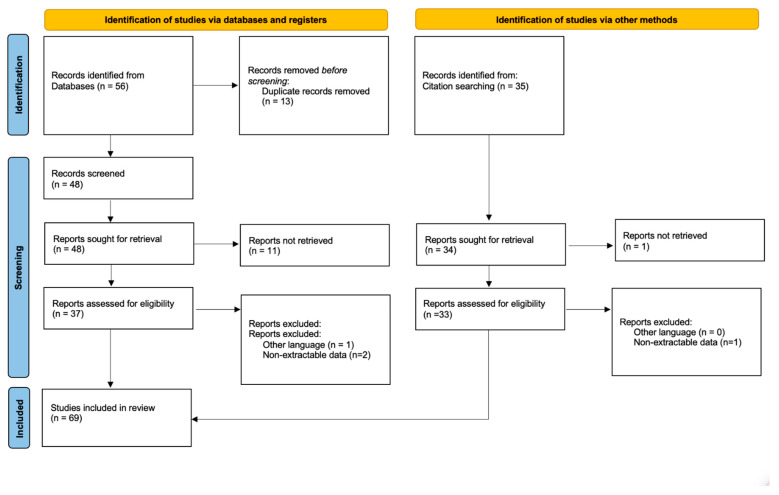
PRISMA flow diagram and the selection of studies.

A total of 56 studies were found through the electronic search and 35 studies were added after the cross-referenced research on the bibliographies of the examined full-text articles (Figure 1).

After a preliminary analysis, a total of 69 studies reporting series of MPs in non-oncologic cases were included in this systematic review (6 prospective studies, 58 retrospective studies, 3 case reports, and 2 retrospective case series).

A total of 2598 MP were retrieved. Among these, 1353 (52.1%) were distal femur (DF) MPs, 941 (36.2%) proximal femur (PF) MPs, 29 (1.1%) proximal tibia (PT) MPs and 259 (10.0%) total femur MPs (Table 1).

Three series reported the combined use of PT and DF MP in a few cases [7,21,22]. Only one case of proximal humerus MP was reported in an aseptic non-union case with proximal humerus arthrosis [79]. Regarding elbow MP, Capanna et al. [80] reported on five revision cases (failed elbow prosthesis or failed osteosynthesis) in a heterogeneous series which included a majority of oncologic MPs. 

The mean age across all studies was 73.2 ± 8.2 years. The mean follow-up period was 39.7 months, ranging between 3 and 88 months. However, not all the included studies reported on the duration of follow-up.

All but three studies detailed the indication to MP. Megaprostheses were most commonly used to treat periprosthetic fractures (1158 cases, 44.6%), in particular in DF (859, 74.2%). Another common indication to implant an MP was a fracture. In 137 cases (5.3%), an MP was used as the primary treatment, whereas in 325 (12.5%) cases it was a salvage procedure to treat a non-union. Megaprostheses were also reported for the treatment of standard prosthesis failure, with 251 (9.9%) cases described after aseptic loosening and 371 (14.3%) to treat a PJI. The majority of MPs in PJI cases were reported in proximal femur (166) compared to DF (83) and total femur (23). Nonetheless, only a few series specifically focus on PJI treatment [13,71,73,75], thus making any evaluation of the efficacy of MP to treat PJI extremely difficult. On the other hand, most of the series were heterogeneous either on the site or the reason to implant an MP. Only five series reported on the use of silver-coated MPs [12,17,26,41,73].

Overall, complications were observed in 513 cases (19.7%) (Table 2). Type I (soft tissue failures) and type IV (infection) were the most frequent (158 and 213, respectively). However, data on infections are difficult to analyze as most of the series did not distinguish between infected/non-infected cases at baseline. Limiting the analysis to series reporting on a single site MP, complications (dislocation in particular) were more commonly observed in TF (34.5%) and PF (26.7%) MPs than in DF MPs (14.7%). 

Functional results were reported only by a few series, with great variability in reported outcome scores. Most of the series focusing on PF used the Harris Hip score (HHS), with a mean value of 72.8, whereas two series reported an Oxford hip score (OHS) of 40 and 30. Only two series reporting only on TF MPs reported a functional assessment, with a mean HHS value of 38.4. These series reported also on knee function in TF with a mean Oxford Knee Score (OKS) of 15.4. Another TF series used the Knee Society Score (KSS) to report functional outcomes (79). Series focused on DF reported a mean OKS of 27.5 and a mean KSS of 77.1.

## 4. Discussion

Several studies on MP for non-tumor reconstruction have been published, but their quality was mainly undermined by heterogeneous populations including different sites and indications. Moreover, some studies reported also on the use of revision arthroplasties mixed with MPs [81,82].

Indication to MP has been described particularly in periprosthetic fractures around a total knee arthroplasty (TKA). Chen et al. [42] compared primary versus secondary DF MP for the treatment of TKA periprosthetic fractures. If ORIF fails, these patients could be revised to a DF MP, but this might expose patients to repeat surgery, and may increase the risk of further complications. Megaprosthesis is a viable treatment option also for DF fractures in the elderly or patients with similarly poor-quality bone. It represents a good alternative to the more commonly used option of distal femoral ORIF/retrograde femoral nails, especially in those patients with radiological evidence of existing osteoarthritis and in the very distal fractures where reconstruction is difficult [52,83]. This can prevent patients from being bedridden and its outcomes such as thrombosis, worsening of dementia, negative impact on independence and autonomy, and the quality of life [84,85]. Similar functional outcomes between ORIF and MP were reported [33,86]. The cost of the implant is higher than that of ORIF but the time to start fully weight-bearing is less. Thus, the higher cost of implants in MP is recouped in the much shorter hospital stay in this procedure [33]. The complication rate of DF MP in non-oncologic cases (9.8%) seems to be generally lower compared to DF MP implanted for oncologic reconstructions (14.6%) [16]. This might be due to several causes which include different ages of populations, comorbidities, and different follow-ups of the studies.

Most PF and TF MPs have been reported as a salvage option for patients with extreme bone loss, once reconstruction with revision stems is no longer feasible, in cases of either aseptic loosening or PJI. Even though they allow for improvement in pain which is comparable to that achieved after revision hip arthroplasty using a conventional hip revision system [61], dislocation is a common complication [64,66,68,87]. Soft tissue failures in PF and TF occurred much more frequently in non-oncologic populations (11.9% and 14.0%, respectively), compared to 5.2% and 8.9%, respectively, in oncologic reconstructions. To reduce the risk of dislocation, attention should be focused on the anatomical reconstruction of muscles such as gluteus and extrarotator of the hip or the iliopsoas. These muscles have to be preserved, where possible, with their bone insertion and linked with the prosthesis in their specific anchoring sites. Moreover, the use of bipolar prostheses, larger femoral heads, constrained liners, or dual mobility cups is advisable [88,89]. Theil et al. [74] reported a high risk of dislocation even among patients treated with dual-mobility acetabular components as part of a two-stage revision for PJI of the hip, with an even higher risk among TF MP than PF MP. The use of bipolar cups had already been suggested by Abdelaziz et al. [90], who observed that revision THA for PJI using a PF MP and a constrained liner or a cemented dual-mobility cup had a comparable dislocation rate with patients treated with a standard THA. However, even though the use of additional constraints (liners or cups) might appear tempting, published results vary tremendously [91,92] and it is unclear whether constrained liners or cups will reduce the risk of instability in patients with a PFR or TFR after a two-stage exchange.

Artificial ligaments can also be used to reduce the dislocation rate [89]. Post-operative care is of paramount importance with immobilization of the limb operated on in abduction for various post-operative durations, and protected weight-bearing thereafter [70].

In the setting of massive segmental defects of the proximal tibia (PT) with loss of collateral ligamentous support and lack of bone to support prosthetic augments or metaphyseal cones or sleeves, a PT MP may create the most biomechanically stable construct. Nonetheless, Henderson et al. [93] found PT MPs to have the highest failure rates of all megaprostheses in oncologic reconstructions, with infection as the leading cause at 16%. It is critical to ensure adequate tissue coverage during closure to prevent infection and enable healing, which may necessitate a flap. Moreover, functional outcomes generally vary based on the extensor mechanism status. In non-oncologic cases, the tibial tubercle can be preserved and healing of the diaphyseal bone has been demonstrated. Thus, it is recommended to preserve the anterolateral column of the proximal tibia including the tubercle when possible to optimize the extension mechanism function. However, only one series specifically focused on PT MPs, thus making any analysis not feasible [13].

In the case of the upper limb, it is impossible to draw any conclusions as there are only two small series available on the topic [79,80]. This lack of evidence for the upper limb is probably due to two main reasons: (1) non-oncological etiologies for massive bone loss are considerably more uncommon; (2) in the case of complex reconstructions, the absence of weight-bearing probably leads to a preference for ORIF or alternative solutions for end-stage scenarios (e.g., proximal humerus permanent spacer, elbow arthrodesis).

Using MPs is undoubtedly an attractive option in end-stage infection scenarios, to avoid amputation. However, concerns over the risk of infection relapse or reinfection remain a reality within the orthopedic community. In cases of post-traumatic septic non-union or prosthetic joint infection (PJI), surgical treatment should be conducted in two steps [7]. In the case of PJI managed with a modular MP, Corona et al. [18] found an overall infection eradication rate of 82.8%, similar to other treatment options. Similar infection control after staged PJI treatment has been reported by Theil et al. [74,75] Despite the greater metallic surface of MP possibly being a significant risk factor for relapse [18], there is the option of performing extensive bone resections—allowing much more aggressive debridement than in normal surgeries—and so eliminating possible osteomyelitis foci that might otherwise have perpetuated the infection.

In cases of revision after infection, the antibiotics added to the cement may have a positive effect on infection control [94,95]. Moreover, even though the cement-free method is particularly advantageous in younger patients [96], in older patients (such as most of those with an MP for non-oncologic indications) with multiple comorbidities, by contrast, the use of the cemented technique can allow immediate full weight-bearing. Nonetheless, the optimal stem fixation for revision remains unknown.

There is a growing trend toward using MPs with surface modifications to reduce the risk of implant colonization. Studies in the literature have reported on three different silver-coated MPs [97], with most of the data coming from oncologic patient series. Fiore et al. [98] highlighted that silver-coated implants are particularly useful in two-stage revisions for infection and in patients with incidental positive cultures at the time of prosthesis implantation [64,98,99,100,101,102]. On the other hand, the results of silver-coated MPs in PJI prevention are extremely heterogeneous. Only a few series described the use of silver-coated MPs in non-oncologic settings [12,17,26,41,73]. Even though they were mainly heterogeneous, including both silver-coated and standard titanium-coated MPs, they were in agreement on the protective role against reinfection when dealing with PJI.

Functional results of megaprotheses seem to be encouraging, in particular in the DF, where similar functional outcomes between ORIF and MP were reported [33,86,103]. On the other hand, functional results in PF and TF can be severely compromised in cases of dislocation or muscle insufficiency. However, functional results in PF MPs used in non-oncologic scenarios seem to be comparable to those observed after revision total hip arthroplasty for a periprosthetic fracture [104] and after hip reimplantation in staged treatments for a PJI [105,106].

There are several limitations to this study. Many of the included series were heterogeneous both in terms of site and reason to implant an MP. There is a real lack of long-term data on MPs in non-oncologic settings, with many series not reporting the outcome. Additionally, many series used different outcome measurements. Moreover, the heterogeneity of most of the series regarding both sites and indications would make any pooled results unsubstantiated. Thus, it is not possible to draw any correlation between the indication to MP and complications.

Megaprosthesis is an attractive option in the management of extreme cases of severe bone loss and prosthetic failure. Benefits of this treatment include relatively short operative times and immediate weight-bearing and resumption of activity. This is highly advantageous in the avoidance of postoperative complications in elderly patients with multiple comorbidities. 

Patients with severe post-traumatic deformities and/or significant bone loss who have had previous septic complications should be considered as an oncologic patient, not because of the disease, but because of the limited therapeutic options available.

## Figures and Tables

**Table 1:** Characteristics of included studies. Site of megaprosthesis and reason to implant. MP: megaprosthesis; PJI: prosthetic joint infection; * Not detailed.

Study	Study Design	Non Oncologic Recontructions (n)	Site				Age (Mean, Years)	Reason to Implant Megaprosthesis					Silver Coating (n)
			Distal Femur (n)	Proximal Tibia (n)	Proximal Femur (n)	Total Femur		Aseptic Loosening (n)	PJI (n)	Fracture (n)	Periprosthetic Fracture (n)	Non Union (n)	
Calori et al. [13]	Retrospective	9		9			68		9				No
Calori et al. [17]	Retrospective	32	13	2	11	6	64	2	5	4		21	32
Corona et al. [18]	Retrospective	29	12		14	3	75		29				No
De Marco et al. [19]	Case series	4	4				77				4		No
Aebischer et al. [20]	Retrospective	306	306				76				306		No
Vitiello et al. [12]	Retrospective	12	6		6		73	1	1	1	5	4	12
Calori et al. [7]	Retrospective	72	31	7	21	13	68	22		5	11	34	No
Fram et al. [21]	Case series	6	2	4			71	1	3	1	1		No
Holl et al. [22]	Retrospective	21	15	6			73	2	5	9		5	No
Kar et al. [23]	Case report	2	2				69					2	No
Toepfer et al. [24]	Retrospective	18				18	78	7			11		No
Toepfer et al. [25]	Retrospective	13				13	73	13					No
Vitiello et al. [26]	Retrospective	23	12		11		73				23		23
Windhager et al. [27]	Retrospective	11	10	1			81				11		No
Zanchini et al. [28]	Retrospective	11	11				86						No
Berend et al. [29]	Retrospective	39	39				76	13	11	11	13	1	No
Keenan et al. [30]	Retrospective	7	7				78			1			No
Springer et al. [31]	Retrospective	26	26				72	8			13	5	No
Stancil et al. [32]	Retrospective	90	90				77			14	58	18	No
Tandon et al. [33]	Retrospective	21	21				78			14	21		No
Chalmers et al. [34]	Retrospective	49	49				76				49		No
Darrith et al. [35]	Retrospective	22	22				76				22		No
Fountain et al. [36]	Retrospective	14				14	64	3	9		2		No
Mortazavi et al. [37]	Retrospective	20	22				70				22		No
Friesecke et al. [38]	Retrospective	96				96	68	31			65		No
Berend et al. [39]	Retrospective	59				59	74	13	14		31		No
Abolghasemian et al. [40]	Retrospective	13	13				77.5						No
Cannon [41]	Retrospective	27	27				*		1		22	4	27
Chen et al. [42]	Retrospective	49	49				74.5				36	13	No
Choi et al. [43]	Case report	1	1				70				1		No
Gan et al. [44]	Retrospective	7	7				76				7		No
Girgis et al. [45]	Retrospective	14	14				82				14		No
Hoellwarth et al. [46]	Retrospective	53	53				80				53		No
Jassim et al. [47]	Retrospective	11	11				81				11		No
Leino et al. [48]	Retrospective	29	29				79				29		No
Matar et al. [49]	Retrospective	30	30				81				30		No
Rahman et al. [50]	Retrospective	17	17				76				17		No
Rao et al. [51]	Retrospective	12	12				78				12		No
Saidi et al. [52]	Retrospective	7	7				80				7		No
Ruder et al. [53]	Retrospective	23	23				80				23		No
Ross et al. [54]	Retrospective	27	27				79				27		No
Haentjens et al. [55]	Retrospective	16			16		78	16					No
Klein et al. [4]	Retrospective	21			21		78				21		No
Parvizi et al. [5]	Retrospective	43			43		74	13	15	3	22	3	No
Shih et al. [56]	Prospective	12			12		59	3	6		3		No
Shoenfeld et al. [57]	Retrospective	19			19		76			10		9	No
Rodriguez et al. [58]	Prospective	97			97		*	*	*	*	*	*	No
Gebert et al. [59]	Retrospective	45			45		62	19	16		9		No
Sewell et al. [60]	Retrospective	15			15		67	4	5		3	3	No
Al-Taki et al. [61]	Retrospective	36			36		73	*	*	*	*	*	No
McLean et al. [62]	Prospective	20			20		72				9	11	No
Dean et al. [63]	Prospective	8			8		67		2	1		5	No
Grammatopoulos et al. [64]	Retrospective	79			79		69		55	24			No
Curtin et al. [65]	Prospective	16			16		75				16		No
Viste et al. [66]	Prospective	44			44		79	17	12		15		No
Khajuria et al. [67]	Retrospective	37			37		80	8	4		8	17	No
De Martino et al. [68]	Retrospective	30			30		64	*	*	*	*	*	No
Fenelon et al. [69]	Retrospective	79			79		78	11	5		55	9	No
Döring et al. [70]	Retrospective	28			28		67	6	11		10	1	No
Logoluso et al. [71]	Retrospective	21			21		68		21				21
Zanchini et al. [72]	Retrospective	39			39		69	15	18		6		No
Dieckmann et al. [73]	Retrospective	49			49		71	29			4	16	41
Theil et al. [74]	Retrospective	70			59	11	73		70				No
Theil et al. [75]	Retrospective	41	41				73		41				No
Sobol et al. [76]	Retrospective	75	75				69	25	23	20	7		No
Barry et al. [77]	Retrospective	22	22				63	6	7	9			No
Wiles et al. [78]	Retrospective	144	144				72	28	40	11	55		No

**Table 2:** Characteristics of included studies. Complications and functional outcomes. HHS: Harris Hip Score; MSTS: Musculoskeletal Tumor Society Scoring System; WOMAC: Western Ontario and McMaster Universities Osteoarthritis Index; OKS: Oxford Knee Score; KSS: Knee Society Score; OHS: Oxford Hip Score; TESS: Toronto Extremity Salvage Score.

Study	Study Design	Non Oncologic Recontructions (n)	Follow-Up (Mean, Months)	Complications				Functional Outcome							
				Type I (n)	Type II (n)	Type III (n)	Type IV (n)	HHS	MSTS	WOMAC	OKS	KSS	Bristol Knee Score	OHS	TESS
Calori et al. [13]	Retrospective	9	18	1						78.2 at 6 months 76.4 at 1 year 74.8 at 18 months					
Calori et al. [17]	Retrospective	32	18	1		1									
Corona et al. [18]	Retrospective	29	48	4			5								
De Marco et al. [19]	Case series	4	3								33.5				
Aebischer et al. [20]	Retrospective	306	24		9	8	10								
Vitiello et al. [12]	Retrospective	12	33												
Calori et al. [7]	Retrospective	72	18	3											
Fram et al. [21]	Case series	6	33												
Holl et al. [22]	Retrospective	21	34		2	2	6								
Kar et al. [23]	Case report	2	12									75			
Toepfer et al. [24]	Retrospective	18	80	5		2	8	40.5			15.5				
Toepfer et al. [25]	Retrospective	13	62	2		1	4	35.4			15.3				
Vitiello et al. [26]	Retrospective	23	24				1								
Windhager et al. [27]	Retrospective	11	40		1		2								
Zanchini et al. [28]	Retrospective	11	23												
Berend et al. [29]	Retrospective	39	24												
Keenan et al. [30]	Retrospective	7	12										80.1		
Springer et al. [31]	Retrospective	26	59			1	5					75.5			
Stancil et al. [32]	Retrospective	90	24			2	2								
Tandon et al. [33]	Retrospective	21	72								28	70			
Chalmers et al. [34]	Retrospective	49	48		6	1	5								
Darrith et al. [35]	Retrospective	22	66	3			1					84			
Fountain et al. [36]	Retrospective	14	89	5			3		17.7						
Mortazavi et al. [37]	Retrospective	20	59			5									
Friesecke et al. [38]	Retrospective	96	59	6		3	12								
Berend et al. [39]	Retrospective	59	56	10			8					79			
Abolghasemian et al. [40]	Retrospective	13	31		1		1					82			
Cannon [41]	Retrospective	27	NR				1					88			
Chen et al. [42]	Retrospective	49	37	5			5								
Choi et al. [43]	Case report	1	12												
Gan et al. [44]	Retrospective	7	44												
Girgis et al. [45]	Retrospective	14	27				1				27				
Hoellwarth et al. [46]	Retrospective	53	12			1									
Jassim et al. [47]	Retrospective	11	33								22.6				
Leino et al. [48]	Retrospective	29	35	3			3								
Matar et al. [49]	Retrospective	30	48	1		3						78			
Rahman et al. [50]	Retrospective	17	34	1		1	1					67.2			
Rao et al. [51]	Retrospective	12	20									72			
Saidi et al. [52]	Retrospective	7	6									74			
Ruder et al. [53]	Retrospective	23	30												
Ross et al. [54]	Retrospective	27	44	1	2	1									
Haentjens et al. [55]	Retrospective	16	60	7	1	3	2								
Klein et al. [4]	Retrospective	21	38	3	1	1	1	71							
Parvizi et al. [5]	Retrospective	43	36	8	4		1	65							
Shih et al. [56]	Prospective	12	68	5	1		4	83							
Shoenfeld et al. [57]	Retrospective	19	44			2	1								
Rodriguez et al. [58]	Prospective	97	38	9	2	3	1	84							
Gebert et al. [59]	Retrospective	45	38	1	2		5	78							
Sewell et al. [60]	Retrospective	15	60					69							
Al-Taki et al. [61]	Retrospective	36	38	3	1	1	1							70	
McLean et al. [62]	Prospective	20	48	3		1	2								68
Dean et al. [63]	Prospective	8	18					71							
Grammatopoulos et al. [64]	Retrospective	79	60	3	3	5	9								
Curtin et al. [65]	Prospective	16	19	2										40	
Viste et al. [66]	Prospective	44	72	6	1		6	68							
Khajuria et al. [67]	Retrospective	37	32	1			3							31	
De Martino et al. [68]	Retrospective	30	60	2	2	2	3								
Fenelon et al. [69]	Retrospective	79	31	12		1	3								
Döring et al. [70]	Retrospective	28	88	8	5	5	6								
Logoluso et al. [71]	Retrospective	21	64	8	2		2								
Zanchini et al. [72]	Retrospective	39	60	2		2	3								
Dieckmann et al. [73]	Retrospective	49	52	6	2	1	2	69							
Theil et al. [74]	Retrospective	70	50	11		2	16								
Theil et al. [75]	Retrospective	41	59				19								
Sobol et al. [76]	Retrospective	75	60	5	10	4	16								
Barry et al. [77]	Retrospective	22	60			1	12								
Wiles et al. [78]	Retrospective	144	60	1	6	2	10					71			

## Data Availability

The data presented in this study are available on request from the corresponding author.
